# Mechanics-based estimation of metabolic cost of locomotion in rehabilitation: A narrative review

**DOI:** 10.1186/s12938-026-01553-2

**Published:** 2026-03-24

**Authors:** Dezhi Jiang, Lauri Stenroth, Rosa Pàmies-Vilà, Parvat Katwal, Suraj Jaiswal, Antti Löppönen, Aki Mikkola, Arend L. Schwab

**Affiliations:** 1 Department of Mechanical Engineering, LUT University, Lappeenranta, Finland; 2 Department of Technical Physics, University of Eastern Finland, Kuopio, Finland; 3 Department of Mechanical Engineering, Universitat Politècnica de Catalunya, Barcelona, Spain; 4 Department of Engineering Sciences, University of Agder, Grimstad, Norway; 5 Gerontology Research Center and the Faculty of Sport and Health Sciences, University of Jyväskylä, Jyväskylä, Finland

**Keywords:** Cost of transport, Joint power, Mechanical cost, Metabolic cost, Physical rehabilitation monitoring

## Abstract

The objective of this paper is to explore methodologies used to estimate the mechanical cost of locomotion and evaluate their validity as indicators of metabolic cost within rehabilitation contexts. Aging societies face rising healthcare costs and fewer workers. Remote monitoring of rehabilitation progress may help address these challenges. Because locomotion mechanics are easier to measure than metabolic cost, understanding whether mechanical cost of transport can serve as a surrogate is of interest. We conducted a systematic search for studies on mechanical work or power and metabolic energy consumption in adults under physical rehabilitation contexts. Abstract, full text screening, and data extraction were performed. Ten articles were included. We found no direct relationship between mechanical cost and metabolic cost in the included studies. However, consistent trends were observed: interventions that provided net positive work generally reduced both mechanical and metabolic costs. Similar trends were found in wheelchair propulsion, where mechanical advantage and power assistance reduced both mechanical cost and metabolic demand for wheelchair users. Rehabilitation progress can be monitored using metabolic cost, and given the observed parallel trends, mechanical cost may also serve as a practical indicator, although further research is needed to establish its validity as a surrogate.

**Supplementary Information:**

The online version contains supplementary material available at 10.1186/s12938-026-01553-2.

## Introduction

In many aging societies, healthcare costs continue to increase while the availability of healthcare professionals declines [1]. Physical rehabilitation [2] is particularly affected, as it typically requires frequent in-person visits and long-term monitoring. To address these challenges, remote and offline monitoring of rehabilitation progress has been proposed as a cost-effective approach that could reduce the burden on healthcare systems and make rehabilitation more accessible [3, 4].

The concept of behavioral energetics [5] provides a useful theoretical framework for understanding why energy efficiency matters in human movement. This emerging field highlights how locomotion patterns are shaped by the natural tendency to minimize energy cost, a preference observed at least in unimpaired individuals [6–8]. It has also inspired new strategies for the rehabilitation of locomotor functions, such as energy-incentivized approaches for individuals after stroke [9, 10], where patients are guided to adopt movement patterns that reduce energy expenditure. Within this perspective, efficiency may be viewed as a potentially informative proxy for the overall functioning of the locomotor system.

Although efficiency-oriented approaches have been widely studied in unimpaired locomotion, their relevance in clinical populations is debated. In stroke survivors and frail older adults, rehabilitation priorities traditionally focus on safety-related outcomes such as fall prevention, gait stability, and symmetry [11–13]. However, inefficient gait is strongly associated with early fatigue, reduced walking endurance, and limited community participation, all of which indirectly increase fall risk and restrict functional independence. From this perspective, movement efficiency should not be viewed as a competing goal, but rather as a complementary indicator of overall locomotor capacity.

This perspective is supported by its application across diverse clinical populations. In chronic stroke, assistive wearable robots have been shown to directly improve metabolic walking efficiency, and thus promote more efficient and balanced walking [14]. Similarly, in knee injury rehabilitation for military personnel, Talbot et al. [15] use work efficiency as the primary outcome to demonstrate the efficacy of their self-managed rehabilitation exercises in combating deconditioning, showing greater improvement than standard rehabilitation alone. For patients with conditions characterized by severe fatigue or low physiological reserve, such as Chronic Obstructive Pulmonary Disease or Myasthenia Gravis, energy management itself becomes a central component of the therapeutic strategy. Rehabilitation programs are designed to incorporate energy conservation principles to prevent exhaustion and safely build capacity, directly aiming to improve quality of life and function [16–18]. However, measurement of metabolic cost is technically demanding, as it usually requires indirect calorimetry or other specialized equipment, making it impractical for widespread or continuous use.

Mechanical aspects of locomotion, such as work and power, can be more readily measured with wearable sensors, including accelerometers and inertial measurement units. In human walking, the mechanical cost can be expressed in different but related ways: as the mechanical power (work performed per unit of time), or as the mechanical cost of transport (CoT; mechanical work spent per unit body mass and distance). Mechanics-based approaches, grounded in Newtonian laws, have long been used to study energy expenditure in humans. Methods range from simple inverted pendulum model [19, 20] to sophisticated multi-segment model [21], and have demonstrated the potential to identify energetically optimal gait patterns. This opens the possibility of using mechanical cost of transport as a surrogate for metabolic cost.

**Fig. 1:**
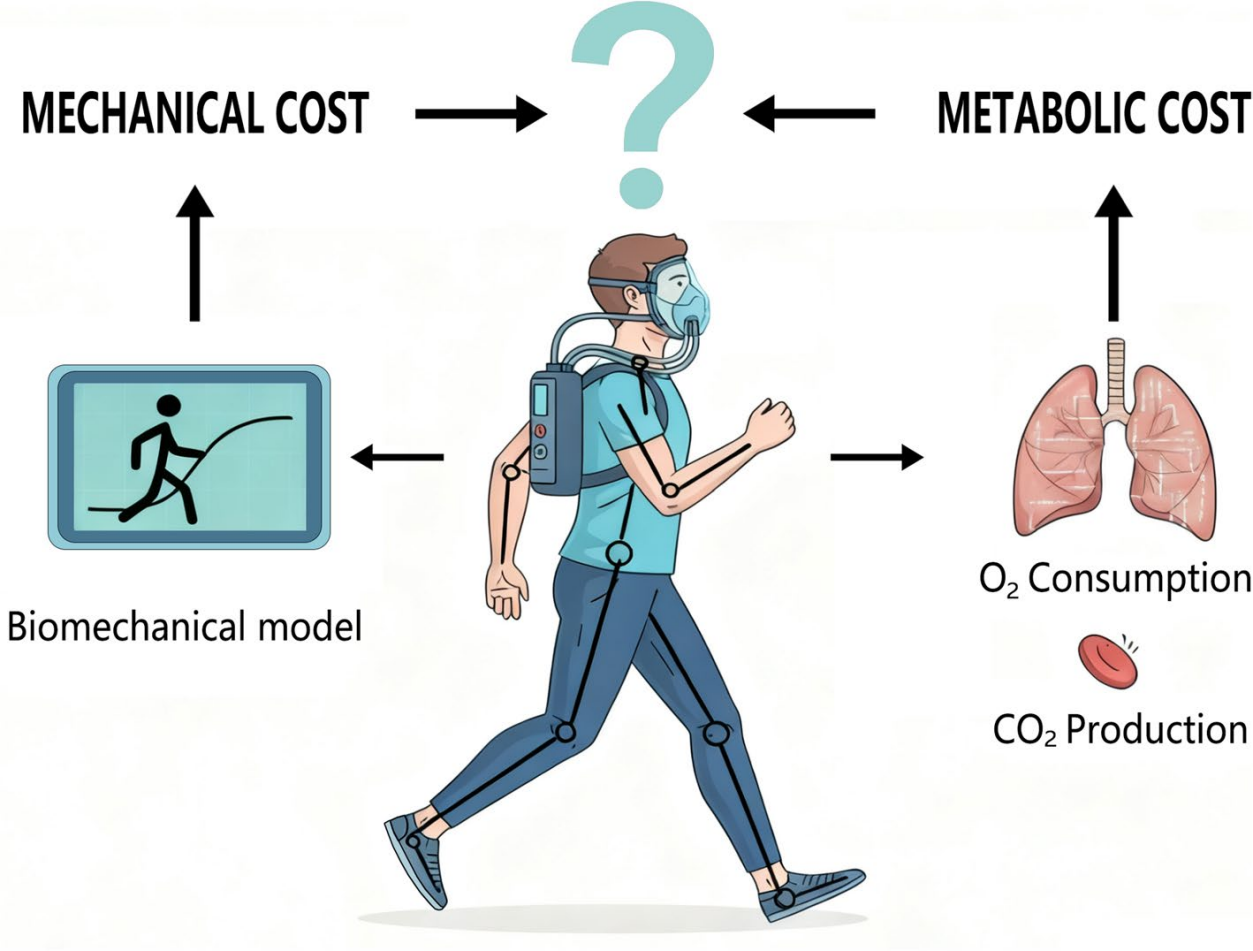
Conceptual illustration of mechanical and metabolic cost assessment during locomotion. The figure shows a person walking while monitored for mechanical and metabolic cost. The question mark emphasizes the potential relationship between mechanical and metabolic costs in physical rehabilitation

Despite the inherent connection between mechanical work and metabolic energy consumption, since the latter is required to generate the former (see Fig 1), their relationship in rehabilitation contexts remains poorly defined. Existing studies suggest that mechanical and metabolic costs often change in parallel, but whether mechanical measures can reliably serve as proxies for metabolic cost is not yet clear.

The objective of this review is therefore to explore the methodologies used to estimate the mechanical cost of locomotion and to evaluate their validity as indicators of metabolic cost in the context of physical rehabilitation. By synthesizing the available evidence, we aim to highlight both the potential and the limitations of mechanics-based estimation as a tool for remote and objective monitoring of rehabilitation progress.

## Method

### Search strategy

A review protocol was established following the Preferred Reporting Items for Systematic Reviews and Meta-Analyses (PRISMA) statement [22]. A comprehensive search was conducted in the Scopus, Web of Science, and PubMed databases. The databases were searched from their inception until September 9, 2025. The search terms included “metabolic energy”, “adult”, “mechanical work”, “musculoskeletal”, and “rehabilitation”, along with synonyms. All terms were combined using the AND operator. The search was restricted to studies published in English with a final publication status. The full search strings are available in Fig. 1.

The initial list of articles was screened in two stages: abstract screening followed by full-text review. The screening was first carried out using Rayyan [23] for a portion of the abstracts and then continued in Covidence [24] for the remaining abstracts and full-text screening. All articles were independently evaluated by at least two reviewers. The PRISMA flow diagram is available in supplementary material. Data extraction was then performed, with two authors independently extracting information from each article. The summary table of data extraction is available in supplementary material. Disagreements were resolved through discussion.

### Inclusion and exclusion criteria

We considered only peer-reviewed studies that investigated both mechanical and metabolic costs in human adults in a rehabilitation context. In this study, physical rehabilitation refers to interventions involving participants who have undergone injury or surgery, studies on injury prevention or with potential rehabilitation applications, and studies involving reduced cost of transport in walking in healthy individuals. Studies involving old participants or pre-existing physical problems were also considered within the scope of rehabilitation. The inclusion criteria are available in supplementary material.

## Definition of key concepts

### Metabolic cost

To maintain consistency in our work, in the context of human walking, we define metabolic cost (expressed per body weight) as the metabolic energy consumed per unit of time (metabolic rate or power) or as the metabolic energy consumed per unit of distance (metabolic CoT). Here, metabolic energy consumption refers to the metabolic energy expended by the body (which is correlated with ATP demand) to perform a specific physical task, such as walking. The metabolic cost is most commonly measured using indirect calorimetry, which estimates energy expenditure based on respiratory gas exchange [25]. Alternatively, direct calorimetry can be used, although it is less practical due to technical complexity. The metabolic cost can also be estimated using simulations based on musculoskeletal energy models [26, 27]. The metabolic rate has been expressed in different units, including milliliters of oxygen uptake per kilogram [28] or joules per kilogram [29]. The metabolic CoT can be expressed as metabolic energy consumption per unit distance traveled [30] or equivalently as metabolic rate divided by walking speed [9]. For a comprehensive overview of metabolic cost definitions, units, and conversions, see Das Gupta et al. [31].

In addition, efficiency reflects the overall function of the locomotory system rather than performance outcomes and is generally defined as the ratio of mechanical work output to metabolic energy input. An example is apparent efficiency, as described in Peyré-Tartaruga et al. [32].

### Mechanical cost

Studies on locomotion quantify mechanical work using different reference systems: COM-based approaches, joint-level inverse dynamics, or device–human interaction measures. These approaches are not directly interchangeable, as they attribute work to different physical entities and rely on distinct assumptions. The terminology of environmental and peripheral work proposed by Kruk et al. [33] is introduced here primarily to harmonize definitions and to clarify how earlier notions of ‘external’ and ‘internal’ work relate to COM-based formulations. This framework is used as a conceptual reference to discuss the strengths and limitations of each method, while the subsequent classification of studies reflects the computational approach actually adopted in the original publications (e.g., joint power, COM work, or device power).

The terms ‘external’ and ‘internal’ work and power are most often found to be inconsistent, as shown by van der Kruk et al. [33]. Internal power may refer to muscular or metabolic work [34, 35], or to the total energy needed to move body segments [36]. External power has also been used to represent different mechanical quantities in sports: frictional power in speed skating [37–39], wheelchair sports [40, 41], and swimming [42], or ergometer output (environmental power) in rowing [43–45] and cycling [46]. In running and walking, external power refers to the rate of change in the center of mass (COM) energy, including kinetic energy [47] and/or gravitational energy [48]. The COM kinematics can be measured with motion capture techniques or can be estimated based on force plate measurements [49, 50]. To reduce confusion caused by these varying definitions, in this review, when separating the power due to the motion of the center of mass (COM) from the power arising from the motion of the body segments relative to the COM for convenience in measurement, we adopt the terminology proposed by van der Kruk et al. [33]. Specifically, the term environmental work is used in place of external work to denote the mechanical work caused by externally applied forces acting on the body (e.g., ground reaction forces). In parallel, the mechanical work done to move body segments relative to the body center of mass is described as peripheral work, replacing the earlier term internal work. Here, environmental work and peripheral work should not be regarded as independent energy measures but rather as descriptive components of the mechanical power balance.

Within the COM-based family of methods, Fenn’s approach [51, 52] has been the most frequently applied strategy to relate mechanical work to metabolic cost, three studies [21, 32, 53] examined the relationship between mechanical work and metabolic cost in walking. This approach, which is based on König’s theorem, defines total mechanical work as the sum of environmental work and peripheral work, both normalized in mass and distance (unit in Joule per kilogram meter). Only positive works were considered to avoid the zero work paradox. The ratio of total mechanical work to metabolic cost is defined as apparent efficiency. Mian et al. [53] investigated the increased metabolic cost of walking (unit in Joule per kilogram meter) in older adults. They found that although older adults had a 31% higher metabolic cost of walking, total mechanical work was only 9% higher and not statistically significant. This resulted in a significantly lower apparent efficiency (−17%) in older adults, suggesting that additional factors, such as increased antagonist muscle coactivation, contribute to the reduced apparent efficiency. Building on this, Peyré-Tartaruga et al. [32] reviewed how mechanical work obtained by the Fenn’s approach has been used to explain the energy cost of human locomotion across modalities (e.g., gradients, low gravity, unsteady conditions) and pathological gaits (e.g., asymmetry, obesity, aging). The authors proposed to improve the estimation of mechanical work by including factors such as frictional internal work and elastic contributions. The recent study by Luciano et al. [21] improved the estimation of mechanical work in multidirectional treadmill walking by incorporating the work against sliding friction, which helped to better account for the increased metabolic cost and reduced the discrepancy in apparent efficiency compared to normal walking.

However, a key limitation of Fenn’s approach is that simply summing environmental and peripheral work can lead to an overestimation of total mechanical work, as demonstrated using a simple 2D two-link model. This overestimation occurs because some forces, such as ground reaction forces, contribute simultaneously to the movement of both the center of mass of the body and the segments [32, 33].

Peyré-Tartaruga et al. [32] noted that there are other methods for calculating the mechanical work in locomotion, such as joint work calculations [54, 55], the cost of generating force [56], and collisional models [57, 58]. Moreover, there is currently no gold standard that fully captures all components of mechanical work and their interdependence. All existing mechanical approaches that are based on body kinematics and external kinetics are also limited by their inability to account for energy consumed during isometric and co-contractions, which do not perform mechanical work but still require metabolic energy.

In contrast, many rehabilitation studies estimate mechanical demand from joint-level power obtained through inverse dynamics, or from the separation between biological and device power in assisted walking. These methods allocate work to anatomical joints or to human–device interaction rather than to the COM and segments. Because these reference systems capture different aspects of the same physical process, their outcomes cannot be directly summed or compared without considering underlying assumptions. For this reason, the present review reports each study according to its original computational framework and subsequently discusses their conceptual compatibility.

## Results

The COM-based mechanical energy method is one of several important approaches for estimating mechanical energy expenditure during human locomotion. However, the included studies in this narrative review did not adopt this approach.

### Estimating metabolic cost of transport from mechanics-based models

Ten articles passed the full-text screening and the relevant data were extracted. Most of the included studies examined walking: [59–66]. Two studies investigated wheelchair propulsion: [67, 68]. Across all included studies, none directly quantify the relationship between mechanical cost and metabolic cost. However, in most cases, the mechanical and metabolic costs showed parallel trends: When an intervention was applied, both the mechanical and metabolic outcomes tended to increase or decrease in the same direction.

Most studies estimated metabolic rate using indirect calorimetry, except the two simulation studies focused on plantarflexor muscles: Sawicki and Khan [66] estimated metabolic power using a mathematical model based on isolated muscle experiments, while Jackson et al. [63] used a modified Umberger model implemented in OpenSim. A summary of reported changes in mechanical and metabolic cost is provided in Table 1 (joint and whole-body level) and Table 2 (muscle-level simulations).

**Table 1:** Mechanical and metabolic cost across interventions

Article	Intervention	Mechanical cost	Metabolic cost
Jackson and Collins [62]	Ankle exoskeleton work +0.25 J/kg/s	−37% positive ankle work rate; +94% total positive ankle work rate	−17%
	Ankle exoskeleton torque +0.18 Nm/kg	−55% positive ankle work rate; −33% total positive ankle work rate	+13%
Jackson et al. [63]$^{*}$	Ankle exoskeleton work $\uparrow$	+137% exoskeleton + tendon positive work rate	$\downarrow$
	Ankle exoskeleton torque $\uparrow$	$\downarrow /=$ (ankle joint work)	$\uparrow$
Panizzolo et al. [65]	Multi-joint soft exosuit power $\uparrow$	−17.2% positive joint work (hip+knee+ankle)	−14.2%
Lerner et al. [60]	Ankle exoskeleton power $\uparrow$	−30% average negative ankle power	−19%
Hu et al. [61]	Foot–ankle exoskeleton stiffness 8.5 N$\cdot$mm/deg (result in power)	Ankle joint work $\downarrow$	−8.19%
Waterval et al. [59]	Ankle–foot orthosis stiffness $\uparrow$	Peak ankle power $\downarrow$	$\downarrow$
McCain et al. [64]	Ankle ROM −29%	−32% peak ankle power	+15%
	Knee ROM −26%	−14% peak ankle power	+9%
	Ankle and knee ROM −39%	−30% peak ankle power	+20%
Woude and den Bot [68]	Mechanical advantage = 0.56	2.3868 kJ/min	23.4 kJ/min
	Mechanical advantage = 0.35	2.4012 kJ/min	20.7 kJ/min
Pradon et al. [67]	Power assistant at wheel $\uparrow$	−32% mechanical power	−45% METs

$\uparrow$ increase, $\downarrow$ decrease, $=$ no change. $^{*}$ Simulation study. Jackson et al. [63]$^{*}$: mechanical cost is reported as unchanged or decreased because joint power was estimated using two different approaches. *ROM* range of motion, *METs* Metabolic equivalents

**Table 2:** Muscle-level mechanical and metabolic costs

Article	Intervention	Mechanical cost	Metabolic cost
Sawicki and Khan [66]$^{*}$	Stiffness of exo-tendon (in parallel with plantarflexors model) = 396 Nm/rad	−80% positive mechanical work of MTU	−30% (ankle PFs)
Jackson et al. [63]$^{*}$	Ankle exoskeleton work $\uparrow$	−77% (muscle fiber work)	−66% (soleus)
	Ankle exoskeleton torque $\uparrow$	+142% (soleus muscle positive work)	$\uparrow$ (soleus)

$\uparrow$ increase, $\downarrow$ decrease. $^{*}$ Simulation study. *MTU* muscle–tendon unit, *PFs* plantarflexors

### Classification of the included studies by mechanical cost category

To help readers, we standardized the terminology for mechanical work and power across studies following the definitions proposed by van der Kruk et al. [33], in which joint power is defined as the mechanical power generated by the human at the joints. In this review, we consistently use the term joint power to denote this biological contribution, even when original articles refer to it as biological joint power [59–63, 65]. The term total power is used to describe the sum of (biological) joint power and device power, where device power refers to the mechanical power delivered by assistive devices such as exoskeletons or orthoses. For instance, Waterval et al. [59] used peak ankle power to describe the combined biological and device contributions, which corresponds to peak total ankle power in our terminology.

A single experimental study focused solely on joint powers without device contributions: McCain et al. [64] investigated the effect of unilateral restrictions of ankle and knee range of motion on walking mechanics and metabolic cost, focusing on joint powers (ankle, knee, hip).

Several experimental studies reported joint and device powers. In these studies, the joint power was obtained by subtracting the device contribution from the total power. Panizzolo et al. [65] investigated the effect of assistive torques from a multi-joint soft exosuit of loaded walking on total positive joint work (sum of hip, knee, and ankle). These torques produced measurable mechanical power at the joints. Waterval et al. [59] investigated the effect of varying dorsal leaf spring ankle–foot orthosis stiffness in patients with calf muscle weakness, and reported total ankle power together with ankle–foot orthosis power (unchanged across stiffness levels). Lerner et al. [60] investigated the effect of a battery-powered ankle exoskeleton on gait in people with cerebral palsy and focused on the average total positive ankle power, the average negative ankle power, and the average positive hip joint power. Hu et al. [61] investigated the effect of an unpowered foot–ankle exoskeleton and reported ankle joint work and power together with beneath-hindfoot work. Although the device is unpowered (no external energy input), it recycles mechanical energy and therefore delivers measurable joint power. Jackson and Collins [62] investigated the independent effects of the net exoskeleton work input and the average exoskeleton torque during walking with a unilateral ankle exoskeleton and focused on the total positive ankle work rate (work divided by the stride period), the contralateral knee work rate, and the total ankle work. In their context, power referred to instantaneous power (torque multiplied by joint angular velocity) expressed in W/kg, whereas work rate was expressed in J/kg/s to distinguish it from instantaneous power.

Two simulation studies were included: both explored the effect of exoskeleton assistance on the plantarflexors. Sawicki and Khan [66] performed a purely simulation-based analysis of the ankle plantarflexor muscle–tendon unit, modeling an exo-tendon in parallel with the plantarflexors and varying its stiffness. Jackson et al. [63] used data from their previous experiment [62] to drive the simulation. Both studies reported the metabolic rate and positive mechanical work of individual muscles, but Jackson et al. [63] further compared ankle joint power estimated from muscle-generated computations with that obtained from inverse dynamics.

Finally, in studies of wheelchair propulsion, Pradon et al. [67] investigated the effect of rear-drive power-assist devices on the biomechanics of the upper limb and the physiological effort during manual wheelchair propulsion and the estimated mechanical power on the hand rim. Van der Woude et al. [68] investigated the effect of different mechanical advantages in lever-propelled wheelchair propulsion on physical strain, efficiency, and mean power output on wheels during steady forward motion.

### Methods and models for mechanical cost

In terms of modeling, standard implementations in Visual3D or OpenSim were the most common, while one [62] used custom-built models from anthropometric data. Most of the included studies estimated the joint powers using an inverse dynamics approach. In this approach, the kinematic data obtained from motion capture and ground reaction forces measured from force plates are combined with the estimated properties of the body segment within a multibody model. This procedure outputs joint moments. The total joint power (or joint power) is then calculated as the product of the joint angular velocity and joint moment. To account for both energy generation and absorption phases, joint work was commonly obtained by integrating the positive and negative portions of the power curve separately over the period of interest.

Within this general approach, Waterval et al. [59] analyzed ankle, knee, and hip powers with the PlugInGait model and also calculated the ankle–foot orthosis power separately. Panizzolo et al. [65] used the multibody model in Visual3D to obtain net joint powers and then integrated positive and negative power to compute biological work. Total joint biological work and power were obtained by adding values in the hip, knee, and ankle. Building on this framework, Hu et al. [61] also isolated ankle joint and beneath-hindfoot powers by subtracting measured exoskeleton power from the total joint power. The work values were then derived as the integrals of positive and negative power in a stride, normalized to body weight. Similarly, Jackson and Collins [62] used a custom multibody model based on published anthropometric data. They first applied inverse dynamics to estimate ankle joint power over periods of positive and negative work and then calculated exoskeleton-side ankle work by subtracting the measured exoskeleton contribution from the total ankle mechanics obtained through inverse dynamics.

Another group of studies implemented inverse dynamics using OpenSim. Lerner et al. [60] scaled a musculoskeletal model and used inverse dynamics to estimate mechanical joint power. The positive and negative mean joint powers during stance were obtained by integrating the respective portions of the joint power time series and dividing by duration, while the net mean power was calculated by integrating the positive and negative portions of the series. McCain et al. [64] used a full-body OpenSim model, with joint powers integrated across ten gait cycles to estimate mechanical work. Jackson et al. [63] applied a generic lower-body musculoskeletal model in OpenSim, driven by electromyography signals and joint angles from their previous experiment [62]. Jackson et al. [63] further compared ankle joint power estimated from muscle-generated computations with that obtained from inverse dynamics. In the muscle-generated ankle joint work computations, the joint moments of the soleus, medial and lateral gastrocnemius, and tibialis anterior were summed and multiplied by ankle angular velocity to obtain muscle-generated power, which was then integrated to yield work.

A separate group of studies focused on wheelchair propulsion, where mechanical power was estimated directly from external force and velocity measurements. In Pradon et al. [67], upper-limb power was computed as the product of force applied at the hand rim and the corresponding angular velocity, both measured with the Smartwheel system. Woude et al. [68] calculated mean wheel power during steady forward motion as the product of drag force and belt velocity. The drag force was measured using a strain-gauged transducer.

### Trends between mechanical and metabolic cost

None of the included studies directly assessed the relationship between mechanical and metabolic costs of transport, but we were able to extract trends between the two.

Assistance that provides net work commonly reduces joint work or power and metabolic cost. However, the effect may be apparent only under certain conditions. Waterval et al. [59] found that the metabolic cost decreased by 15.3−18.6% in all levels of ankle-foot orthosis stiffness of the dorsal leaf spring compared to shoes alone. The most efficient stiffness reduced the metabolic cost by 21% relative to shoes alone and by 10.7% compared to the least efficient stiffness. They reported that increasing stiffness reduced maximal ankle dorsiflexion angle (stabilizing gait), but decreased peak ankle power (decrease forward propulsion) by 0.09 W/kg for every 1 Nm/deg increase in stiffness. The effect is also visible in the supplementary data: peak ankle power declined from 1.54 W/kg at K1 (lowest stiffness) to 1.14 W/kg at K5 (highest stiffness), whereas group-level metabolic cost reached its lowest mean value (4.21 J/kg/m) at the stiffness level K2. Here, the positive work of ankle-foot orthosis (0.06 J/kg/m) did not change across stiffness conditions.

In Lerner et al. [60], untethered ankle exoskeleton assistance increased total average positive power (sum of device and joint) by 44% and reduced average negative ankle power by 30%. Ankle assistance also reduced positive hip joint power requirements by 29%, and the metabolic cost of transport decreased by 19%. Since average positive ankle power did not significantly change across conditions, these results suggest that powered ankle assistance enhanced biological function instead of simply replacing it.

In Hu et al. [61], the net metabolic rate decreased with medium assistance from an unpowered foot–ankle exoskeleton by 8.19% but not at low or high levels. Ankle joint work and power, plantarflexion moments, and beneath-hindfoot work were reduced during push-off and heel-strike, while exoskeleton work and power increased with assistive magnitude.

Panizzolo et al. [65] reported that the exosuit-powered assistance reduced the sum of positive joint work of the hip, knee, and ankle joints from 1.28 to 1.06 J kg compared to the unpowered condition and from 1.22 to 1.06 J kg compared to the unpowered condition with equivalent mass removed. The net metabolic power was reduced by 14.2% compared to the unpowered condition and by 7.3% compared to the unpowered condition with the equivalent mass removed. They further observed that, on average, participants saved 1.8 J of metabolic energy for every 1 J of exosuit positive mechanical work delivered.

The muscle–tendon modeling study Sawicki and Khan [66] reported that, as exoskeleton stiffness increased, plantarflexor muscle–tendon unit work and power decreased linearly. Tendon recoil is reduced to almost zero at 70% or more of normal ankle joint stiffness. The metabolic cost versus exoskeleton stiffness relationship showed a minimum in moderate stiffness, where plantarflexor cost was reduced by about 30% (of unassisted walking), corresponding to an overall reduction in metabolic cost of approximately 8%.

The other group further showed that, while assistance providing net work reduced joint work and metabolic cost, a pure torque-support condition without net work instead led to an increase in metabolic cost. Jackson and Collins [62] found that increasing exoskeleton work from the zero work to high work condition (net exoskeleton work rate: 0.25 J/kg/s) reduced ankle work rate from 0.21 to 0.095 J/kg/s (−37%, meaning human contribution reduced), which is consistent with the observed reduction of metabolic cost by 17%; while increased the total positive ankle work rate from 0.17 to 0.31 J/kg/s (94%) showing that the overall mechanical output at the joint was enhanced. The contralateral knee work rate (both negative and positive) also reduced by 44% and 48%, respectively, showing ankle interventions affect whole-body coordination. However, increasing torque support from zero torque to high torque condition (average torque: 0.18 Nm/kg, no net work) increased metabolic cost by 13%, decreased the total positive ankle work rate from 0.21 to 0.14 J/kg/s (33%), and increased contralateral knee work during double support, indicating an added metabolic cost for the human as the contralateral limb compensated for reduced ankle push-off of the exoskeleton side.

Jackson et al. [63] found that providing plantarflexion torque with no net work increased the positive muscle fiber work (late stance) of the soleus by 232% and the positive work rate by 142%, while the combined exoskeleton–tendon–soleus work remained unchanged. In contrast, providing net positive ankle work decreased the soleus positive work rate by 73% and muscle fiber work by 77%, while combined exoskeleton–tendon work increased by 137% and total system work by 72%. Net exoskeleton work also reduced the estimated soleus metabolic rate by 66%.

Some studies questioned the direct relationship between mechanical cost and metabolic cost. Jackson et al. [63] showed that ankle joint work is not a reliable predictor of energy consumption of muscle. With increasing exoskeleton torque, soleus muscle fiber work increased, while ankle joint work estimated from muscle-generated computations remained unchanged, but ankle joint work estimated through inverse dynamics decreased. Therefore, the finding suggested the importance of muscle–tendon mechanics for designing assistive devices.

McCain et al. [64] reported that, relative to baseline (ankle range of motion: $24.1^{\circ }$; peak ankle power: 1.94 W/kg), peak ankle power decreased by 32%, 14%, and 30% when ankle, knee, and combined restriction of ankle and knee reduced range of motion to $16.97^{\circ }$, $17.96^{\circ }$, and $14.61^{\circ }$, respectively. The metabolic cost increased by 15%, 9%, and 20% under these same conditions, respectively. No significant correlation was found between total positive joint power and net metabolic power. Changes in metabolic rate were therefore not explained by changes in mechanical power alone. The combined restriction of ankle and knee range of motion showed the highest metabolic cost despite the lower total positive joint power. Hip power increased in ankle restriction condition, but did not correspond to metabolic savings. These results suggest that compensatory mechanics (hip hike, circumduction) can drive an increased metabolic cost rather than just joint work. Unlike previous studies, increases in metabolic cost were not directly proportional to increases in mechanical power, indicating that traditional work efficiency models may not fully account for metabolic cost in walking under restrictions. The findings highlight the need for alternative metrics beyond positive joint power to estimate metabolic demand in impaired gait.

Unlike the reductions in joint powers that were accompanied by increased metabolic cost, likely reflecting compensatory mechanics in healthy participants [64], Waterval et al. [59] found that using optimal efficient ankle-foot orthosis stiffness for persons with non-spastic calf muscle weakness reduced both peak ankle power and metabolic cost. This contrasting reduction in metabolic cost may be explained by the orthosis stabilizing gait through limiting excessive dorsiflexion, which is a key contributor to elevated metabolic cost in neuromuscular patients.

For wheelchair propulsion, van der Woude et al. [68] examined the effects of varying mechanical advantage through gear ratios in a lever-propelled wheelchair and showed that mechanical advantage strongly influenced mechanical efficiency and metabolic cost; specifically, lower mechanical advantages were associated with reduced oxygen cost and energy expenditure. Here, a lower mechanical advantage (e.g., MA4 = 0.35) means relatively more force is required but each stroke moves the wheelchair further, whereas a higher MA (e.g., MA1 = 0.56) requires less force but more strokes. At MA4, the authors reported a higher mechanical efficiency of 11.6% compared to 10.2% at MA1. This corresponds to a slightly higher mechanical power output at the wheels ($2.4012~\text {kJ/min}~\approx ~40.0~\text {W}$ vs. $2.3868~\text {kJ/min}~\approx ~39.8~\text {W}$), while requiring less metabolic power ($20.7~\text {kJ/min}~\approx ~345~\text {W}$ vs. $23.4~\text {kJ/min}~\approx ~390~\text {W}$).

In the latter work, Pradon et al. [67] found that compared to manual wheelchair use, when the rear-drive power assist device was operated in full power mode (around 6 km/hour on flat and smooth ground), mechanical power from the users decreased by about 32% (from 10.6 to 7.2, unit was not stated), and both oxygen consumption and metabolic equivalents decreased by about 45% (from 11.8 to 6.5 ml/min/kg and from 3.4 to 1.9, respectively).

## Discussion

Methodologically, the included studies are united by their use of joint-level analysis, focusing on the attribution of power and biological versus device contributions.

In all included studies, we did not find a direct relationship between mechanical and metabolic cost, but we observed parallel trends under certain conditions: when interventions delivered net positive device work, both mechanical and metabolic costs tended to decrease.

Among the walking studies, when assistive devices provided net positive work, both positive ankle joint power and metabolic cost decreased, indicating a reduced human contribution. At the same time, the total power (sum of device and human contributions) increased, suggesting that the human could move more easily with assistance. In contrast, when torque was applied without delivering work, metabolic cost often increased despite reductions in positive ankle joint power, likely due to compensatory mechanics, as in Jackson et al. [62, 63], Panizzolo et al. [65], and Hu et al. [61]. A similar pattern was observed when joint range of motion was restricted as shown in McCain et al. [64]. However, as reported by Waterval et al. [59], this compensatory penalty reflected in elevated metabolic cost may not be evident in neuromuscular patients, although peak ankle power for push-off was decreased.

The relationship between mechanical and metabolic costs is not always straightforward, as mechanical measures alone cannot fully account for increased metabolic demand. Beyond measurement limitations, physiological factors contribute to the disparity between mechanical and muscle power, as noted previously by van der Kruk et al. [33]. This has also been shown outside the studies included in our review. For example, Mian et al. [53] concluded that, elevated metabolic cost in older versus young adults in walking could not be explained by differences in whole-body mechanical work but was attributed to antagonist muscle coactivation for stability. Similarly, Luciano et al. [21] found that, in multidirectional treadmill walking, additional effort to overcome sliding friction meant that whole-body mechanical work underestimated metabolic demand. These examples highlight that additional energetic demands may arise that are not captured by conventional joint or whole-body mechanical work estimates.

Based on the reviewed literature, there seems to be a lack of studies investigating direct relationships between the mechanics-based and metabolic cost of transport. Specifically, there is a lack of information on the utility of simple mechanical models based on easily measured kinematic data, such as a single accelerometer or IMU, in estimating the metabolic cost of locomotion. Therefore, future research directions include the experimental validation of mechanical quantities in varied contexts. For example, using the mechanical cost to assess the gait energetic economy or metabolic cost in clinical populations, such as total knee arthroplasty patients, at various time points—before surgery, immediately after surgery, and at extended follow-ups to better understand the implications of mechanical quantities on physical rehabilitation.

## Conclusion

- We found no direct relationship between mechanical cost and metabolic cost in the included studies.
- Despite this, consistent trends were observed: interventions that provided net positive work generally reduced mechanical cost and metabolic cost.
- In wheelchair propulsion, a lower mechanical advantage was observed alongside a lower metabolic and mechanical cost. Power assistance at the wheel was found to reduce both mechanical power and oxygen consumption for the user.
- Rehabilitation progress can be monitored using metabolic cost, and given the observed parallel trends, mechanical cost may also serve as a practical indicator.
- The use of wearable sensors and mechanics-based models offers opportunities for a continuous, remote, and objective assessment of gait and mobility in clinical populations.
- Future research could simultaneously apply measurements from different mechanical frameworks (e.g., joint-level and COM-based methods) with gold-standard metabolic energy to validate their agreement, while also exploring their applicability across diverse rehabilitation scenarios and refining models to account for compensatory strategies and non-mechanical energy demands.

## Supplementary Information

Supplementary material 1. Search strings

Supplementary material 2. PRISMA flow diagram

Supplementary material 3. Summary table: Detailed table summarizing the extracted data of the included studies

Supplementary material 4. Inclusion criteria

## Data Availability

The datasets supporting the conclusions of this article are included within the article and its additional file(s).
