# Supplementary material for: Mechanics-based estimation of metabolic cost of locomotion in rehabilitation: A narrative review
Source: Biomed Eng Online. 2026 Mar 24;25:67. doi: 10.1186/s12938-026-01553-2 (PMC13137497; doi:10.1186/s12938-026-01553-2)
Supplement: Supplementary file 1 — Supplementary material 1. Search strings [file 12938_2026_1553_MOESM1_ESM.docx]

1. Scopus

( TITLE-ABS-KEY ( "Oxidative Phosphorylation" OR "energy metabolism" OR "energy expenditure" OR "metabolic cost" OR "energy cost" OR "energetic cost" OR "metabolic consume*" OR "energy consume*" OR "caloric expenditure*" OR "metabolic power*" ) ) AND ( TITLE-ABS-KEY ( "Human*" OR "Adult*" OR "Old*" OR "Elderly*" OR "Aging*") ) AND ( TITLE-ABS-KEY ( "Mechanical Work" OR "cost of transport*" OR "biomechanical work" OR "mechanical energy*" OR "biomechanic*" OR "musculoskeletal mechanic*" OR "force production*" OR "muscle work*" OR "kinetic energy*" OR "kinematic*" OR "power*" ) ) AND ( TITLE-ABS-KEY ( "musculoskeletal" AND "rehabilitation*") ) AND ( LIMIT-TO ( LANGUAGE , "English" ) ) AND ( LIMIT-TO ( PUBSTAGE , "final" ) )

1. Web of science

(TI=("Oxidative Phosphorylation" OR "energy metabolism" OR "energy expenditure" OR "metabolic cost" OR "energy cost" OR "energetic cost" OR "metabolic consume*" OR "energy consum*" OR "caloric expenditure*" OR "metabolic power*")

OR AB=("Oxidative Phosphorylation" OR "energy metabolism" OR "energy expenditure" OR "metabolic cost" OR "energy cost" OR "energetic cost" OR "metabolic consume*" OR "energy consum*" OR "caloric expenditure*" OR "metabolic power*")

OR AK=("Oxidative Phosphorylation" OR "energy metabolism" OR "energy expenditure" OR "metabolic cost" OR "energy cost" OR "energetic cost" OR "metabolic consume*" OR "energy consum*" OR "caloric expenditure*" OR "metabolic power*"))

AND

(TI=("Human*" OR "Adult*" OR "Old*" OR "Elderly*" OR "Aging*")

OR AB=("Human*" OR "Adult*" OR "Old" OR "Elderly*" OR "Aging*")

OR AK=("Human*" OR "Adult*" OR "Old" OR "Elderly*" OR "Aging*"))

AND

(TI=("Mechanical Work" OR "cost of transport*" OR "biomechanical work" OR "mechanical energy*" OR "biomechanic*" OR "musculoskeletal mechanic*" OR "force production*" OR "muscle work*" OR "kinetic energy*" OR "kinematic*" OR "power*")

OR AB=("Mechanical Work" OR "cost of transport*" OR "biomechanical work" OR "mechanical energy*" OR "biomechanic*" OR "musculoskeletal mechanic*" OR "force production*" OR "muscle work*" OR "kinetic energy*" OR "kinematic*" OR "power*")

OR AK=("Mechanical Work" OR "cost of transport*" OR "biomechanical work" OR "mechanical energy*" OR "biomechanic*" OR "musculoskeletal mechanic*" OR "force production*" OR "muscle work*" OR "kinetic energy*" OR "kinematic*" OR "power*"))

AND

(TI=("musculoskeletal" AND "rehabilitation")

OR AB=("musculoskeletal" AND "rehabilitation")

OR AK=("musculoskeletal" AND "rehabilitation"))

1. Pubmed:

((Oxidative Phosphorylation[Title/Abstract] OR energy metabolism[Title/Abstract] OR energy expenditure[Title/Abstract] OR metabolic cost[Title/Abstract] OR energy cost[Title/Abstract] OR energetic cost[Title/Abstract] OR metabolic consume*[Title/Abstract] OR energy consume*[Title/Abstract] OR caloric expenditure*[Title/Abstract] OR metabolic power*[Title/Abstract]))

AND

((Human*[Title/Abstract] OR Adult*[Title/Abstract] OR Old OR Elderly*[Title/Abstract] OR Aging*[Title/Abstract]))

AND

((Mechanical Work[Title/Abstract] OR cost of transport*[Title/Abstract] OR biomechanical work[Title/Abstract] OR mechanical energy*[Title/Abstract] OR biomechanic*[Title/Abstract] OR musculoskeletal mechanic*[Title/Abstract] OR force production*[Title/Abstract] OR muscle work*[Title/Abstract] OR kinetic energy*[Title/Abstract] OR kinematic*[Title/Abstract] OR power*[Title/Abstract]))

AND

((musculoskeletal[Title/Abstract] AND rehabilitation[Title/Abstract]))

1. IEEE Xplore (In abstract)

"Oxidative Phosphorylation" OR "energy metabolism" OR "energy expenditure" OR "metabolic cost" OR "energy cost" OR "energetic cost" OR "metabolic consume*" OR "energy consume*" OR "caloric expenditure*" OR "metabolic power*"

AND

"Human*" OR "Adult*" OR "Old*" OR "Elderly*" OR "Aging*"

AND

"Mechanical Work" OR "cost of transport*" OR "biomechanical work" OR "mechanical energy*" OR "biomechanic*" OR "musculoskeletal mechanic*" OR "force production*" OR "muscle work*" OR "kinetic energy*" OR "kinematic*" OR "power*"

AND

"musculoskeletal" AND "rehabilitation*"
