# Supplementary material for: Mechanics-based estimation of metabolic cost of locomotion in rehabilitation: A narrative review
Source: Biomed Eng Online. 2026 Mar 24;25:67. doi: 10.1186/s12938-026-01553-2 (PMC13137497; doi:10.1186/s12938-026-01553-2)
Supplement: Supplementary file 2 — Supplementary material 2. PRISMA flow diagram [file 12938_2026_1553_MOESM2_ESM.docx]

**Identification of studies via databases and registers**

Records removed *before screening*:

Duplicate records removed (n = 19)

Records marked as ineligible by automation tools (n = 0)

Records removed for other reasons (n = 0)

Records identified from*:

Databases (n = 4)

Scopus n = 81

Web of Science n = 21

Pub Med n = 7

IEEE Xplore n = 3

**Identification**

Records screened

(n = 93)

Records excluded**

(n = 39)

Reports sought for retrieval

(n = 54)

Reports not retrieved

(n = 0)

**Screening**

Reports assessed for eligibility

(n = 54)

Reports excluded:

No mechanical cost (n = 27)

No metabolic cost (n = 14)

Wrong population (n = 3)

Studies included in review

(n = 10)

Reports of included studies

(n = 10)

**Included**
