# Supplementary material for: Mechanics-based estimation of metabolic cost of locomotion in rehabilitation: A narrative review
Source: Biomed Eng Online. 2026 Mar 24;25:67. doi: 10.1186/s12938-026-01553-2 (PMC13137497; doi:10.1186/s12938-026-01553-2)
Supplement: Supplementary file 3 — Supplementary material 3. Summary table: Detailed table summarizing the extracted data of the included studies [file 12938_2026_1553_MOESM3_ESM.pdf]

Article	Intervention	population	health condition	mechanical model	mechanical measure	Method of mechanical measure	Metabolic measure	Method of metabolic measure	main finding
Jackson 2015	ankle exoskeleton (work)	7M + 1F	Healthy	A custom multibody model based on published anthropometric data	Exoskeleton work, Exoskeleton-side ankle work, Contralateral knee work, Center-of-mass push-off, collision, and rebound work (separated positive and negative phases). (Exoskeleton power: W/kg; work rate: J/kg/s)	Exoskeleton work: Measured exo torque × ankle angular velocity → integrate over stride → divide by stride time.<br>Exoskeleton-side ankle work: Inverse dynamics for total; subtract exo power to get biological; integrate positive/negative portions.<br>Contralateral knee work: Inverse dynamics on unassisted knee; integrate positive/negative power.<br>Center-of-mass work: From limb-specific GRFs and COM velocity; integrate push-off, collision, rebound phases.	Net metabolic rate (W/kg)	indirect calorimetry (Oxycon Mobile; CareFusion, San Diego, CA)	Increasing exoskeleton work from the Zero Work condition to the High Work condition, reduced metabolic cost (17%) and reduced positive ankle work rate (37%) while reducing negative and positive contralateral knee work rate by 44 and 48%, respectively. In contrast, increasing torque support from the Zero to the High Torque condition, raised metabolic cost (13%), decreased positive ankle joint work rate (55%), and increased contralateral knee work during double-support.
Panizzolo 2016	Multi-joint soft exosuit (work)	7	Healthy	Visual 3D Multibody model	Total joint biological positive work and power (sum of hip, knee, and ankle); Total joint biological negative work. (power:W/kg; work: J/kg)	The exosuit moment was subtracted from the net joint moment, then multiplied by joint velocity to get biological power. Biological work was obtained by integrating positive and negative power over time. Total joint biological work and power obtained by summing values across hip, knee, and ankle.	Net metabolic power (W/kg)	indirect calorimetry (K4b2, Cosmed, Rome, Italy)	The exosuit reduced the total joint positive biological work when comparing the EXO_ON condition (1.06 J/kg) with respect to the EXO_OFF condition (1.28 J/kg) and to the EXO_OFF_EMR (device mass removed) condition (1.22 J/kg). Net metabolic power in the EXO_ON condition (7.5 W/kg) was 7.3 % and 14.2 % lower than in the EXO_OFF_EMR condition (7.9 W/kg) and in the EXO_OFF condition (8.5 W/kg), respectively. Metabolic savings were proportional to mechanical work delivered, with 1.8 J of metabolic energy saved per 1 J of exosuit mechanical work.
Lerner 2019	untethered wearable ankle exoskeleton (power)	Not mentioned.	Ambulatory individuals with CP	Scaled Opensim musculoskeletal model	Total positive ankle joint power; average negative ankle power; average positive hip joint power (power:W/kg)	Mechanical Joint Power: the product of ankle angular velocity and joint moment. Positive and negative mean joint power during stance were obtained by integrating the positive or negative portions of the joint power time series and dividing by the duration. Net Mean Power: integrating across both the positive and negative portions of the joint power time series.	Metabolic cost of transport (J/kg/m).	indirect calorimetry (K5, Cosmed); Brockway's standard equation	External assistance increases average total positive ankle power (44%), reduced average negative ankle power (30%), reduced average positive hip joint power requirements (29%). Metabolic cost of transport reduced by 19%. The results indicate that powered ankle assistance augmented, rather than simply replaced, biological function.

Hu 2022	unpowered exoskeleton	8M	Healthy	Visual 3D Multibody model	Hip, knee, and ankle joint power ; Beneath-hindfoot power; Exoskeleton ankle power; Biological ankle joint work (power:W/kg; work: J/kg)	Hip, knee, and ankle joint power: inverse dynamics in Visual3D by combining filtered GRF. Beneath-hindfoot power: Computed with a unified deformable segment analysis. Exoskeleton ankle power was computed from measured rope force and device geometry; exoskeleton beneath-hindfoot power was estimated from its work ratio to total beneath-hindfoot work. Biological ankle and beneath-hindfoot powers were obtained by subtracting the exoskeleton component from the total, and work values were the integrals of the positive/negative portions over a stride, normalized to body weight. (MATLAB)	net metabolic rate (W/kg).	indirect calorimetry (Indirect spirometry: Jaeger Oxycon Mobile)	Metabolic cost of walking decreased at medium assistance (8.19 %) but not at low or high levels. Biological ankle joint work and power, plantarflexion moments, and beneath-hindfoot work were reduced during push-off and heel-strike, while exoskeleton work and power increased with assistive magnitude.
Sawicki 2016 (simulation)	elastic ankle exoskeleton	Not involved.	Not involved.	a simple lumped uniarticular musculoskeletal model of the plantarflexors operating in parallel with an elastic "exo-tendon."	mechanical power (W) of the muscle–tendon unit (MTU) and its elements: contractile element (CE) and series elastic element (SEE)	Mechanical power was calculated as the product of force and velocity Eq (6). Work was calculated by integrating power Eq (7).	Metabolic power (W) by the lumped plantarflexor, Compensatory metabolic cost (J).	mathematical model.	As EXO stiffness increased, mechanical work and power generated by the lumped plantarflexor muscle–tendon unit, contractile element, and series elastic element decreased overall. Total energy cost of muscles working has a minimum (about 30% lower than no EXO) at a given exoskeleton stiffness value.
Jackson 2017 (simulation)	ankle exoskeleton	7M + 1F	Healthy	A generic lower-body musculoskeletal model	Ankle joint power (W/kg)	Ankle joint power was calculated as the product of ankle joint moment and angular velocity. The joint moment was obtained using OpenSim's inverse dynamics tool.	Metabolic rate for each muscle (W/kg)	modified version of Umberger's muscle metabolics model implemented in OpenSim	Providing net exoskeleton work at the ankle reduced metabolic rate, whereas providing only torque increased metabolic rate. Across exoskeleton work conditions, Total estimated soleus metabolic rate decreased by 66%; positive work rate of the soleus muscle decreased by 73%.
Waterval 2019	ankle-foot orthosis	21M + 16F	Various neuromuscular disorders with non-spastic calf muscle weakness.	Vicon Plugin Gait multibody model	Ankle peak power (total); AFO generated power (W/kg)	The kinematics and kinetics (powers) of the ankle, knee, and hip, were obtained from combined motion capture and force plate data using the PlugInGait model.<br>with peak ankle power taken from the stance phase. AFO power was calculated separately from the product of AFO stiffness, deflection angle, and angular velocity.	mean energy consumption (J/kg/sec); walking energy cost (J/kg/m)	indirect calorimetry (Cosmed K4B2, Rome, Italy)	The more the AFO increases ankle peak power, the more it reduces energy cost. However, this association only holds in specific comparisons (e.g., best vs. no AFO) and not across all stiffness levels. The practical and causal implications remain unclear.

McCain 2021	walking under restrictions (ankle, knee)	7M + 8F	Healthy	OpenSim Multibody model	Peak restricted limb ankle power, restricted limb knee power absorption, Average positive hip joint power (W/kg).	Joint powers computed using inverse dynamics in OpenSim. Mechanical Power at joints computed and integrated across 10 gait cycles to estimate mechanical work.	Net metabolic rate (W/kg).	indirect calorimetry (Cosmed K5, USA)	Restricted ankle increased restricted limb average positive hip power, but decreased in the restricted knee and restricted ankle-and-knee conditions. Restricted motions in all 3 cases above were associated with a significant increase in net metabolic rate.<br>For walking under restrictions, the study found no significant correlation between mechanical joint power and metabolic power, and concluded that the metabolic consequences highlight the potential energetic benefit of targeting ankle function during rehabilitation.
VanDerWoude 1997	lever-propelled wheelchair	10M	Healthy	Product of force and velocity	Mean power (W) output at the wheels to maintain steady forward motion	Mean power = dragforce *belt velocity<br>Drag force was measured using a strain-gauged force transducer.	Total metabolic energy expenditure (kJ/s) determined with respiratory exchange ratio and oxygen uptake (L/min).	indirect calorimetry (gas analyser Ox-4, Mijnhardt).	Varying mechanical advantage (MA) through gear ratios in a lever-propelled wheelchair significantly affected mechanical efficiency (ME) and energy expenditure (En), with lower MAs showed lower oxygen cost and En.
Pradon 2022	manual wheelchair	17M + 5F	Spinal Cord injury	Product of force and velocity	Mechanical power at hand rim. Unit was not stated.	Mechanical power was computed from the tangential torque applied at the hand rim and the corresponding angular velocity, both measured via the Smartwheel system.	Metabolic equivalents; oxygen consumption (ml/min/kg)	indirect calorimetry (MetaMax 3B system)	Power assistance on upper-limb reduced: oxygen consumption (45%), METs (45%), mechanical power (34%).
