# Supplementary material for: Mechanics-based estimation of metabolic cost of locomotion in rehabilitation: A narrative review
Source: Biomed Eng Online. 2026 Mar 24;25:67. doi: 10.1186/s12938-026-01553-2 (PMC13137497; doi:10.1186/s12938-026-01553-2)
Supplement: Supplementary file 4 — Supplementary material 4. Inclusion criteria [file 12938_2026_1553_MOESM4_ESM.docx]

**Inclusion criteria**

- Q1: Is the study focused on human subjects?

It is enough that you can assume based on the context that it is human related, with ‘Yes’.

- Q2: Are the subjects aged 18 or older?

If it doesn't specify all participants are kids, we can assume it is (with ‘Unsure’).

If the participants are “adolescent”, use unsure.

- Q3: Is the study related to rehabilitation applications?

If the abstract mentioned their study have potential application in rehabilitation area, or the participants undergone injury or surgery, then use ‘Yes’.

If the abstract state that their study has potentials for injury prevention and rehabilitation, use “unsure”. Same apply in metabolic cost part.

Reducing the cost of transport of walking, (without disease) for example, can be assumed as a rehabilitation application on its own. Using ‘Unsure’.

However, improving working performance (with exoskeleton) for young and healthy individuals does not belong to rehabilitation.

If there are pre-existing problems, such as the worker is under the influence of back pain or elderly, then it is related to rehabilitation.

- Q4: Does the abstract mention the estimation of mechanical energy?

If explicitly quantifying mechanical energy, answer ‘Yes’.

If they did not quantify the mechanical energy, but they have everything needed (such as force generation, torque production) to compute the mechanical work, we can use ‘Unsure’.

It is acceptable to have only part of the mechanical work/power, such as joint work/power. (full text screening)

- Q5: Does the abstract mention the estimation of metabolic energy?

Include all studies that measure metabolic energy, even if it uses surrogate measures like oxygen consumption.

About conflict in abstract, for example, if the method part only mention measuring pressure, but in collusion part it mentioned reducing metabolic cost, we can use “unsure”.

It is acceptable if the metabolic cost is obtained through simulation. (full text screening)
